# Hypoxia-preconditioned mesenchymal stem cells attenuate bleomycin-induced pulmonary fibrosis

**DOI:** 10.1186/s13287-015-0081-6

**Published:** 2015-05-20

**Authors:** Ying-Wei Lan, Kong-Bung Choo, Chuan-Mu Chen, Tsai-Hsien Hung, Young-Bin Chen, Chung-Hsing Hsieh, Han-Pin Kuo, Kowit-Yu Chong

**Affiliations:** Division of Biotechnology, Graduate Institute of Biomedical Sciences, College of Medicine, Chang Gung University, Tao-Yuan, Taiwan Republic of China; Department of Preclinical Sciences, Faculty of Medicine and Health Sciences and Centre for Stem Cell Research, Universiti Tunku Abdul Rahman, Selangor, Malaysia; Department of Life Sciences, National Chung Hsing University, Taichung, Taiwan Republic of China; Agricultural Biotechnology Center, National Chung Hsing University, Taichung, Taiwan Republic of China; Rong-Hsing Translational Medicine Center, National Chung Hsing University, Taichung, Taiwan Republic of China; Institute of Biotechnology, National Taiwan University, Taichung, Taiwan Republic of China; Graduate Institute of Clinical Medical Sciences, College of Medicine, Chang Gung University, Tao-Yuan, Taiwan Republic of China; Department of Thoracic Medicine, St Paul’s Hospital, Taoyuan, Taiwan Republic of China; Department of Thoracic Medicine, Ton-Yen General Hospital, Hsinchu, Taiwan Republic of China; Department of Thoracic Medicine, Pulmonary Disease Research Center, Chang Gung Memorial Hospital, Taipei, Taiwan Republic of China; Department of Medicine, College of Medicine, Chang Gung University, Tao-Yuan, Taiwan Republic of China; Department of Medical Biotechnology and Laboratory Science, College of Medicine, Chang Gung University, Tao-Yuan, Taiwan Republic of China; Molecular Medicine Research Center, College of Medicine, Chang Gung University, Tao-Yuan, Taiwan Republic of China

## Abstract

**Introduction:**

Idiopathic pulmonary fibrosis is a progressive diffuse parenchymal lung disorder of unknown etiology. Mesenchymal stem cell (MSC)-based therapy is a novel approach with great therapeutic potential for the treatment of lung diseases. Despite demonstration of MSC grafting, the populations of engrafted MSCs have been shown to decrease dramatically 24 hours post-transplantation due to exposure to harsh microenvironments. Hypoxia is known to induce expression of cytoprotective genes and also secretion of anti-inflammatory, anti-apoptotic and anti-fibrotic factors. Hypoxic preconditioning is thought to enhance the therapeutic potency and duration of survival of engrafted MSCs. In this work, we aimed to prolong the duration of survival of engrafted MSCs and to enhance the effectiveness of idiopathic pulmonary fibrosis transplantation therapy by the use of hypoxia-preconditioned MSCs.

**Methods:**

Hypoxic preconditioning was achieved in MSCs under an optimal hypoxic environment. The expression levels of cytoprotective factors and their biological effects on damaged alveolar epithelial cells or transforming growth factor-beta 1-treated fibroblast cells were studied in co-culture experiments *in vitro*. Furthermore, hypoxia-preconditioned MSCs (HP-MSCs) were intratracheally instilled into bleomycin-induced pulmonary fibrosis mice at day 3, and lung functions, cellular, molecular and pathological changes were assessed at 7 and 21 days after bleomycin administration.

**Results:**

The expression of genes for pro-survival, anti-apoptotic, anti-oxidant and growth factors was upregulated in MSCs under hypoxic conditions. In transforming growth factor-beta 1-treated MRC-5 fibroblast cells, hypoxia-preconditioned MSCs attenuated extracellular matrix production through paracrine effects. The pulmonary respiratory functions significantly improved for up to 18 days of hypoxia-preconditioned MSC treatment. Expression of inflammatory factors and fibrotic factor were all downregulated in the lung tissues of the hypoxia-preconditioned MSC-treated mice. Histopathologic examination observed a significant amelioration of the lung fibrosis. Several LacZ-labeled MSCs were observed within the lungs in the hypoxia-preconditioned MSC treatment groups at day 21, but no signals were detected in the normoxic MSC group. Our data further demonstrated that upregulation of hepatocyte growth factor possibly played an important role in mediating the therapeutic effects of transplanted hypoxia-preconditioned MSCs.

**Conclusion:**

Transplantation of hypoxia-preconditioned MSCs exerted better therapeutic effects in bleomycin-induced pulmonary fibrotic mice and enhanced the survival rate of engrafted MSCs, partially due to the upregulation of hepatocyte growth factor.

## Introduction

Idiopathic pulmonary fibrosis (IPF) is a chronic and progressive form of interstitial lung disease characterized by an intricate cytokine network and abnormal deposition of mesenchymal cells [1,2]. To date, there are no effective and approved treatments for IPF [3], making IPF the most life-threatening idiopathic disease with a median survival of about 3 years from initial diagnosis.

Mesenchymal stem cell (MSC)-based cell therapy is a potential therapeutic approach for the treatment of various lung diseases [4,5]. Several studies have demonstrated that MSCs home to sites of lung injury, an event also associated with reduced tissue damage, inhibited production of proinflammatory mediators, decreased extracellular matrix collagen deposition, and generally contributed to tissue repair [6,7]. In addition, MSCs secrete paracrine factors with anti-inflammatory, anti-apoptotic and anti-fibrotic functions [8,9]. Despite reported success, it was demonstrated that the amount of engrafted MSCs decreased dramatically after 24 hours of transplantation due to exposure to toxic and oxidative microenvironments [10]. Recent studies involving overexpressing anti-oxidant [11,12] or anti-apoptotic genes or growth factors in engrafted stem cells have shown improved survival following transplantation [13]. In addition, cells, tissues or whole animals may be preconditioned by sub-lethal exposure to selected stresses to induce prior expression of cytoprotective genes before subsequent lethal challenges [14]. Cellular preconditioning may include exposure of cells to physiological stimuli such as hypoxia, heat shock, small-molecule pharmacological agents, cytokines, growth factors or biophysical stimuli. Alternatively, cellular preconditioning may be achieved by genetic manipulation by overexpression of anti-apoptotic proteins, chemokine receptors, growth factors or pro-survival genes prior to transplantation [15-18].

Adopting appropriate preconditioning strategies may provide a simple yet effective way of promoting survival and regenerative properties, and also the tissue repair capability of transplanted cells in stem cell-based therapy [19]. Recent studies have also shown that short-term exposure of MSCs to sub-lethal hypoxia before transplantation enhances cell survival and downregulation of apoptosis-related pathways [19,20]. Furthermore, hypoxic preconditioning also induces the expression of pro-survival markers [21], chemoattractants [22] and growth factors involved in cell proliferation, anti-oxidation, anti-apoptosis and angiogenesis [23] in MSCs. Hypoxic preconditioning and stem cell transplantation have been extensively studied in a number of organs and tissues in relation to diseases to enhance therapeutic effects; the studies have included ischemic diseases such as stroke and myocardial infarction [19,24-26], traumatic brain injuries [27], diabetes mellitus [28], inflammatory bowel disease [29], and acute kidney [22,30] and liver injuries [31]. However, there have been no published studies on using hypoxia-preconditioned MSCs (HP-MSCs) in the treatment of pulmonary fibrosis.

In this work, we investigated if hypoxic preconditioning of MSCs would lead to comprehensive regulation that promotes cell survival, enhances cytoprotective and growth factor action, and attenuates extracellular matrix (ECM) production. These effects were anticipated to result in inhibited inflammatory response, fibrotic factor production, and significant improvements in lung functions in a lung fibrosis animal model.

## Material and methods

### Chemicals

Bleomycin sulfate from *Streptomyces verticillus* was obtained from Sigma-Aldrich (St Louis, MO, USA). The c-Met inhibitor PHA-665752 was purchased from Santa Cruz Biotechnology, Inc. (Dallas, TX, USA).

### Cell lines

MSCs were isolated from the bone marrow of C57BL/6 female mice. MSCs were purchased from Life Technologies (GIBCO mouse C57BL/6 MSCs; Carlsbad, CA, USA) following the guidelines of Good Manufacturing Practices for medical derivatives, Code of Federal Regulations Title 21 (21 CFR), Part 820 of the US Food and Drug Administration regulation. The female murine alveolar epithelial cell line (MLE-12; CRL-2110) was purchased from American Type Culture Collection (Manassas, VA, USA). Both cell lines were maintained in Dulbecco’s modified Eagle’s medium/Ham’s Nutrient Mixture F-12 (Life Technologies) supplemented with 10% fetal bovine serum (Life Technologies), 2 mM L-glutamine (Life Technologies) and 1% penicillin/streptomycin (Life Technologies). Human 14-week male embryonal lung cell line (MRC-5; BCRC-60023) was purchased from Bioresource Collection and Research Center (Hsinchu, Taiwan). MRC-5 cells were maintained in Eagle’s minimal essential medium (Life Technologies) supplemented with 10% fetal bovine serum (Life Technologies) and 1% penicillin/streptomycin (Life Technologies), and were incubated at 37°C in a 5% CO_2_ incubator.

### Viral production and viral transduction

Virus stocks were prepared by co-transfecting the pLenti6/v5-GW/lacZ plasmid (Life Technologies) with three packaging plasmids, pMDLg/pRRE, CMV-VSVG and RSV-Rev, into 293 T cells following the method of Chen and colleagues [32]. The viral supernatants were harvested 36 to 48 hours later, filtered and centrifuged at 20,000 × *g* for 90 minutes. The viral titer was determined by the method of end-point dilution through counting the number of infected red cells at ×100 magnification under a fluorescence microscope 96 hours after infection to 293 T cells. Titer in transducing units was computed as follows: (TU)/mL = (the numbers of red fluorescent cells) × (dilution factor)/(volume of virus solution). Titers of the viral particles were quantified by HIV-quantification enzyme-linked immunosorbent assay kit. MSCs were seeded in 12-well plates and the cells were transduced with an equal ratio of viral particles of pLenti6/v5-GW/lacZ virus particle and the stably transduced cells were designated as β-Gal-MSCs.

### Hypoxic preconditioning

MSCs were grown to confluency and were changed to fresh complete medium before hypoxia treatment using a finely-controlled ProOx-C-chamber system (Biospherix, Redfield, NY, USA) for 24 hours. The oxygen concentration in the chamber was maintained at 1.5% with a residual gas mixture composed of 5% carbon dioxide and balanced nitrogen. Normoxia-treated MSCs used as a control were cultured in 95% atmospheric air and 5% CO_2_ for 24 hours. Conditioned medium was collected from MSCs cultured in normoxic or hypoxic conditions.

### Measurement of mitochondrial membrane potential

Mitochondrial membrane potential was assessed using a sensitive fluorescent probe JC-10 (Enzo Life Sciences Inc., Farmingdale, NY, USA). MSCs were incubated with JC-10 (1 μM) at 37°C for 30 minutes. JC-10 is capable of selectively entering into mitochondria, and reversibly changes its color from green (JC-10 monomeric form) to orange (JC-10 aggregate form) as membrane potentials increase. Both colors can be detected using flow cytometers (FACSCalibur; BD Biosciences, San Jose, CA, USA).

### Mesenchymal stem cells and MRC-5 co-culture assay

MSCs were plated at a density of 1 × 10^5^ cells/well and MRC-5 cells were plated at a density of 2 × 10^5^ cells/well in transwells (BD Biosciences) and 6-well culture plates (BD Biosciences), respectively, and the cells were cultured overnight. MSCs were then treated with the indicated oxygen concentrations in hypoxic treatment for 24 hours. MRC-5 cells were treated with or without 2.5 ng/mL transforming growth factor (TGF)-β1 (Sino Biological Inc., Beijing, China) for 24 hours. After removing the medium, hypoxia-pretreated MSCs in transwells were co-cultured with the MRC-5 cells for 24 hours. The MRC-5 cells were harvested for detection of fibronectin mRNA expression level by quantitative real-time RT-PCR.

### PHA665752 treatment

MRC-5 cells were plated at a density of 2 × 10^5^ cells/well in 6-well culture plates (BD Biosciences) and cells were treated with or without indicated concentrations of PHA665752 (Sigma-Aldrich) and 2.5 ng/mL TGF-β1 (Sino Biological) for 24 hours. After removing the medium, hypoxia-pretreated MSCs in transwells were co-cultured with the PHA665752-treated MRC-5 cells for 24 hours. The MRC-5 cells were harvested for detection of fibronectin mRNA expression level by quantitative real-time RT-PCR.

### Cell proliferation and viability test

MSCs were plated at a density of 5 × 10^4^ cells/well in a 12-well culture plate (BD Biosciences) and incubated overnight to allow adhesion. The cells were then treated with the indicated concentrations of oxygen for 24 hours. The cells were detached by trypsinization and were counted in triplicate using a Countess™ automated cell counter (Life Technologies). Cell viability was measured using 0.4% (w/v) trypan-blue (Sigma-Aldrich) exclusion and the Countess™ software.

### Cell viability MTT assay

MLE-12 cells were plated at a density of 1 × 10^5^ cells/well in a 24-well culture plate (BD Biosciences) and were cultured to confluency. The cells were treated with bleomycin (BLM; 13.5 μM) in the presence of normoxia-preconditioned MSC (NP-MSC)- or HP-MSC-conditioned medium for 48 hours. The cell viability was then detected using the MTT method at a wavelength of 540 nm by VERSAMax spectrophotometry (Molecular Devices, Sunnyvale, CA, USA) as previous described [33].

### Short hairpin RNA and transient transfection

The hepatocyte growth factor (HGF) short hairpin RNA (shRNA) clone (TRCN0000336131; GGTAAAGGAGGCAGCTATAAA) targeted at the mouse HGF transcript was purchased from the National RNAi Core Facility (Academia Sinica, Taipei, Taiwan). MSCs were plated at a density of 1 × 10^5^ cells/well in a 24-well plate and were transfected with 2.5 μg HGF shRNA plasmid premixed with 2.5 μl Lipofactamine™ LTX (Life Technologies) for 24 hours. At the end of the incubation, MSCs were exposed to hypoxia for 24 hours. Following hypoxic preconditioning, cells were used in co-culture assays.

### Annexin V/propidium iodide double-staining assay

At 1 hour post-H_2_O_2_ (Sigma-Aldrich) treatment (0.5, 1, and 2 μM), cell viability of NP-MSCs and HP-MSCs were detected by Annexin V/propidium iodide (PI) staining (Strong Biotech Co., Taipei, Taiwan). Briefly, 1 × 10^5^ cells were labeled with 1 μL Annexin V and PI in a binding buffer for 15 minutes. The cells were analyzed using a flow cytometer (FACSCalibur; BD Biosciences). The percentage of cell numbers in each quadrant was calculated using CELLQUEST software (BD Biosciences).

### RNA isolation and quantitative real-time RT-PCR

Total RNA was prepared from the cell lines and was treated with DNase I (New England BioLabs, Ipswich, MA, USA). RNAs were reverse transcribed into cDNAs at 42°C for 60 minutes using Moloney Murine Leukemia Virus Reverse Transcriptase (Life Technologies). After the oligo (dT)-primed reverse transcription reaction, quantitative real-time RT-PCR was performed using LightCycler 480 SyberGreen I Master Mix and LightCycler® 480 Instrument (Roche, Mannheim, Germany) as previously described [34]. Sequences of the mouse gene-specific primers used and for the human fibronectin are listed in Table 1. For normalization, the GADPH mRNA level of each RNA preparation was determined. Relative gene expression was determined by the ΔΔCt method, where Ct is threshold cycle. The relative mRNA levels were normalized to the mRNA level of the reference GADPH gene. The melting curve of the amplification product was always checked to ensure a single clean peak that represented good-quality quantitative real-time RT-PCR data.

**Table 1:** **Primer sequences**

**Gene**	**Primer sequence**
HO-1	Forward: AAGCCGAGAATGCTGAGTTCA
	Reverse: GCCGTGTAGATATGGTACAAGGA
VEGFa	Forward: CTGTGCAGGCTGCTGTAAC
	Reverse: ACAGTGATTTTCTGGCTTTGTTC
BAX	Forward: CGGCGAATTGGAGATGAACTG
	Reverse: GCAAAGTAGAAGAGGGCAACC
Bcl-2	Forward: TACCGTCGTGACTTCGCAGAG
	Reverse: CAGGCTGAGCAGGGTCTT
CAT	Forward: CCTGACATGGTCTGGGACTT
	Reverse: CAAGTTTTTGATGCCCTGGT
HGF	Forward: GGCTGAAAAGATTGGATCAG
	Reverse: TGGTTCTTGGTGTCATTGTTCCT
HIF-1α	Forward: GGACGATGAACATCAAGTCAGCA
	Reverse: GGAATGGGTTCACAAATCAGCAC
EPOR	Forward: GGCTCCGAAGAACTTCTGTG
	Reverse: CCAGGAGCACTACTTCATTG
Fibronectin	Forward: CCCACCGTCTCAACATGCTTAG
	Reverse:CTCGGCTTCCTCCATAACAAGTAC
Pro-IL-1β	Forward: GCTCATCTGGGATCCTCTCC
	Reverse: CCTGCCTGAAGCTCTTGTTG
IL-6	Forward: CCACTTCACAAGTCGGAGGCTTA
	Reverse: GCAAGTGCATCATCGTTGTTCATAC
Col III	Forward: GTTCTAGAGGATGGCTGTACTAAACACA
	Reverse: TTGCCTTGCGTGTTTGATATTC
CTGF	Forward: ACCTGGAGGAAAACATTAAGAAGG
	Reverse: AGCCCTGTATGTCTTCACACTG
LacZ	Forward: CCGTTGATGTTGAAGTGGC
	Reverse: CTAATCCGAGCCAGTTTACCC
β-Actin	Forward:GCGAGAAGATGACCCAGATC
	Reverse:CCAGTGGTACGGCCAGAGG
GAPDH	Forward: AGAGACGGCC GCATCTTCTT
	Reverse: CCGTTCACACCGACCTTCAC

BAX-2, Bcl-2-associated X protein; Bcl-2, B-cell lymphoma 2; CAT, catalase; Col III, collagen type III; CTGF, connective tissue growth factor; EPOR, erythropoietin receptor; HGF, hepatocyte growth factor; HIF-1α, hypoxia-inducible factor 1α; HO-1, heme oxygenase-1; IL, interleukin; VEGF, vascular endothelial growth factor.

### Western blot analysis

Total cellular proteins were isolated from cell lines by the PRO-PREP™ Protein Extraction Solution (Intron Biotechnology, Kyonggi-do, Korea). Western blot analysis was performed as described previously [35]. Briefly, 25 or 50 μg total protein from cell lysates or conditioned media was loaded onto each lane and the proteins were separated in SDS-PAGE (Bio-Rad Laboratories, Hercules, CA, USA). After electrophoresis, the resolved proteins were transferred to PVDF membrane (Millipore, Billerica, MA, USA). The membranes were blocked with 5% skimmed milk powder (Anchor, Kowloon, Hong Kong) in phosphate-buffered saline (PBS)-Tween (PBS containing 0.1% Tween-20 (both Sigma-Aldrich)) for 1 hour and probed overnight with the following antisera at appropriate dilutions: anti-heme oxygenase 1 (HO-1; MBL International, Woburn, MA, USA; 1:1,000), anti-fibronectin, anti-HGF, anti-vascular endothelial growth factor (VEGF) and anti-interleukin (IL)-1β (Santa Cruz Biotechnology; 1:1,000), primary antibodies detecting pan-AKT or phospho-AKT S473 (Cell Signaling Technology, Beverly, MA, USA; 1:2,000), and anti-β-actin (Millipore; 1:10,000) antisera in PBS-Tween. Identification of each protein was achieved with the Western Lighting Plus Reagent (Perkin Elmer, Waltham, MA, USA) using an appropriate horseradish peroxidase-conjugated secondary antibody (Jackson Immuno Research Laboratories, West Grove, PA, USA). Protein levels in the western blot analysis were detected and quantified by the LAS-3000 chemiluminescence detection device (Fujifilm, Valhalla, NY, USA). To adjust for loading differences, the optical density of each protein was normalized to that of the β-actin band.

### Animal model of bleomycin-induced lung fibrosis

Eight-week old male C57BL/6JNarl mice were purchased from the National Laboratory Animal Center (Taipei, Taiwan). The mice were maintained in an air-conditioned animal facility under constant temperature and humidity conditions with a 12:12 light-dark cycle and were allowed *ad libitum* diet and drinking water. Mice were randomly picked to different groups and there were at least six or more mice in each group. All experimental procedures were approved by the Institutional Animal Care and Use Committee of the Chang Gung University. Bleomycin sulfate (Sigma-Aldrich) stock was prepared by dissolving in sterile PBS at 10 mg/mL and storing in small aliquots at 4°C. Mice were anesthetized by isoflurane (Abbott Laboratories, Abbott Park, IL, USA) inhalation and BLM was instilled intratracheally at 1.5 mg/kg body weight in 50 μL sterile PBS. All animals received intratracheal instillation of either BLM or PBS on day 0. On day 3, mice were randomly selected for intratracheal injection (5 × 10^5^ cells in 50 μl PBS) of normoxia- or hypoxia-pretreated MSCs, or just PBS. On day 4 and day 18 after stem cell transplantation, animals were placed in the whole-body plethysmograph tanks for analysis of pulmonary functions. At each time point, the mice were sacrificed by an overdose of 2.5% avertin (Sigma-Aldrich).

### Noninvasive measurement of pulmonary functions by whole-body plethysmography

The baseline enhanced respiratory pause (Penh) in unrestrained conscious mice was measured by whole-body plethysmography (Buxco Electronic Inc., Wilmington, NC, USA). Mice were placed in the chamber and allowed to equilibrate for approximately 10 minutes. According to the manufacturer’s instructions, Penh is calculated by the formula Penh = (Te/Rt-1) × (PEF/PIF), where Te is expiratory time (seconds); Rt is relaxation time (seconds), defined as the time of “volume” decay to 35% of the total expiratory pressure signal (area under the box pressure signal at expiration); PEF is peak expiratory flow (ml/second); and PIF is peak inspiratory flow (ml/second). Penh reflects changes in the waveform of the box pressure signal during both inspiration and expiration, and combines these changes with the timing comparison of early and late expiration during the animal’s spontaneous breathing. Penh was measured for 3 minutes for each experiment.

### Lung morphometry

Left lungs were fixed with 10% formalin (Macron Fine Chemicals™, Avantor Performance Materials, Center Valley, PA, USA) and were embedded in paraffin sections before staining with hematoxylin and eosin (Sigma-Aldrich) according to standard protocols. Briefly, 4-μm serial step sections were taken along the longitudinal axis of the lobe. The fixed distance between the sections was calculated to allow systematic sampling of 10 sections across the whole lobe. The severity of lung fibrosis was assessed by measuring the Masson’s trichrome staining [36]. The Ashcroft score was used for quantitative histological analysis of fibrotic changes induced by BLM. Five fields within each lung section were observed, and the score of fibrosis ranged from 0 (normal lung) to 8 (total fibrous obliteration of the field) [37].

### Immunohistochemical staining of β-gal

Immunohistochemistry was performed to evaluate engraftment of transplanted β-Gal-MSCs *in vivo*. Paraffin-fixed sections were microwave-heated (750 W, three 5-minute cycles) in 0.01 M citrate (pH 6.0) or 1-mM EDTA (pH 8.0) solution. Immunostaining was performed with a 1:1,000 dilution of the anti-β-Galactosidase antibody (ab9361; Abcam Inc, Cambridge, MA, USA) using the Dako-REAL, Alkaline-Phosphatase/RED detection system (Dako, Glostrup, Denmark). Hematoxylin was used for counterstaining according to the manufacturer’s protocol. The stained sections were scanned by the HistoFAXS (TissueFAX plus; TissueGnostics, Vienna, Austria).

### Collagen staining

The Sircol collagen assay (Biocolor Ltd., Belfast, UK), a dye-binding method designed for the analysis of acid and pepsin-soluble collagens, was used to detect the amounts of collagenous protein in lung tissues. The stained tissue homogenate released Sircol red dyes by an alkali reagent (1 N NaOH, Sigma-Aldrich) and measured by VERSAMax spectrophotometry (Molecular Devices). The amounts (μg) of collagenous proteins in each tissue were determined by OD at 550 nm.

### Statistical analysis

Data are either shown as individual data points in a vertical scatter dot plot, with a line to indicate the mean, or as bar graphs showing mean ± standard deviation (SD). Comparisons between two groups were analyzed using the two-tailed Student’s *t-*test. For multiple comparisons, one-way analysis of variance analysis was used, followed by the Tukey’s *post hoc* test for analyzing parametric data. All statistical analyses were performed using Graph Pad Prism (GraphPad Software, Inc., San Diego, CA, USA). *P < 0.05, **P < 0.01 and ***P < 0.001 are considered statistically significant.

## Results

### Hypoxic preconditioning upregulates expression of cytoprotective and regenerative factors in mesenchymal stem cells

**Figure 1:**
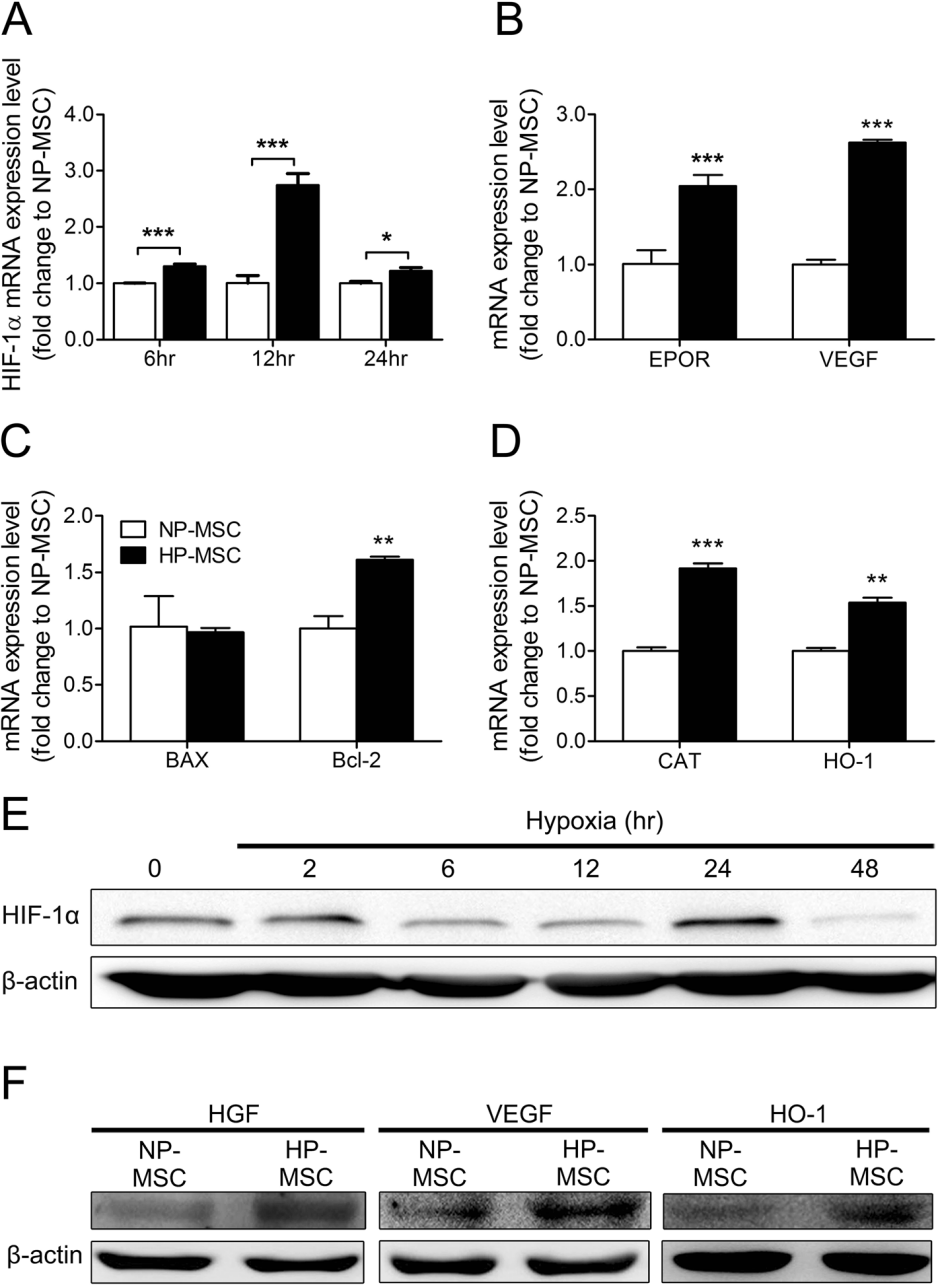
Effects of hypoxic preconditioning on cytoprotective and regenerative gene expression in hypoxia-preconditioned mesenchymal stem cells. Quantitative real-time RT-PCR was performed to examine the relative expression levels of mRNAs. **(A)** Hypoxia-induced factor (HIF)-1α in mesenchymal stem cells (MSCs) after 6 to 24 hours exposure under 1.5% oxygen conditions. **(B)** The HIF-1α downstream target genes, erythropoietin receptor (EPOR) and vascular endothelial growth factor (VEGF), **(C)** pro- and anti-apoptotic factors, B-cell lymphoma 2-associated X protein (BAX) and B-cell lymphoma 2 (Bcl-2), and **(D)** anti-oxidants, catalase (CAT) and heme oxygenase 1 (HO-1), in normoxia-preconditioned MSCs (NP-MSCs; 20% O_2_ for 24 hours) and hypoxia-preconditioned MSCs (HP-MSCs; 1.5% O_2_ for 24 hours). **(E)** Western blot analysis was performed to detect the expression level of HIF-1α in MSCs after 2 to 48 hours incubation under the 1.5% oxygen condition. **(F)** Comparison of protein levels of HGF, VEGF, and HO-1 in NP-MSCs (20% O_2_ for 24 hours) and HP-MSCs (1.5% O_2_ for 24 hours) in western blot analysis. Mouse β-actin was used as the normalization control. (A-D) Open and filled bars indicate NP-MSCs and HP-MSCs, respectively. Values were normalized to β-actin and are expressed relative to the respective control group. *P < 0.05, **P < 0.01, ***P < 0.001.

To optimize hypoxic conditions for preconditioning MSCs, the time course of hypoxia-inducible factor (HIF)-1α expression during 48 hours exposure to a 1.5% O_2_ concentration was followed. The mRNA expression level of HIF-1α showed a gradual increase at 6 hours and reached peak level at 12 hours of hypoxic exposure (2.74 ± 0.2, peak expressed as relative gene expression changes; Figure 1A). A peak protein expression level of HIF-1α was observed after 24 hours of exposure (Figure 1E). Results showed that expression of the hypoxia marker, HIF-1α, was upregulated significantly in hypoxia- compared with normoxia-treated MSCs (Figure 1A). HIF-1α plays a predominant role in the upregulation of survival and anti-oxidant genes and also growth factors [26,38]. To characterize the HP-MSCs, the expression levels of representative cytoprotective and regenerative factors were determined using quantitative real-time RT-PCR and western blot analysis. Erythropoietin receptor, one of the regulators of HIF-1α-mediated cell survival [19], showed increased levels in HP-MSCs (2.04 ± 0.15, expressed as relative gene expression changes; Figure 1B). Moreover, the multifunctional cytoprotective gene, HGF, and the proangiogenic gene, VEGF (2.62 ± 0.04, expressed as relative gene expression changes), were also significantly overexpressed in HP-MSCs (Figure 1B). The anti-apoptotic gene, B-cell lymphoma 2(Bcl-2), was also significantly upregulated (1.61 ± 0.03, expressed as relative gene expression changes) under hypoxic conditions; however, there were no significant differences in the expression of B-cell lymphoma 2-associated X protein (BAX) between normoxic and hypoxic cells (Figure 1C). Furthermore, low oxygen tension also upregulated the levels of the anti-oxidant genes, catalase (1.92 ± 0.05, expressed as relative gene expression changes) and HO-1 (1.54 ± 0.05, expressed as relative gene expression changes) in HP-MSCs (Figure 1D). Upregulated expression of HGF, VEGF and HO-1 was further confirmed in western blot analysis (Figure 1F).

### Hypoxic preconditioning stabilizes the mitochondrial membrane potential in mesenchymal stem cells

**Figure 2:**
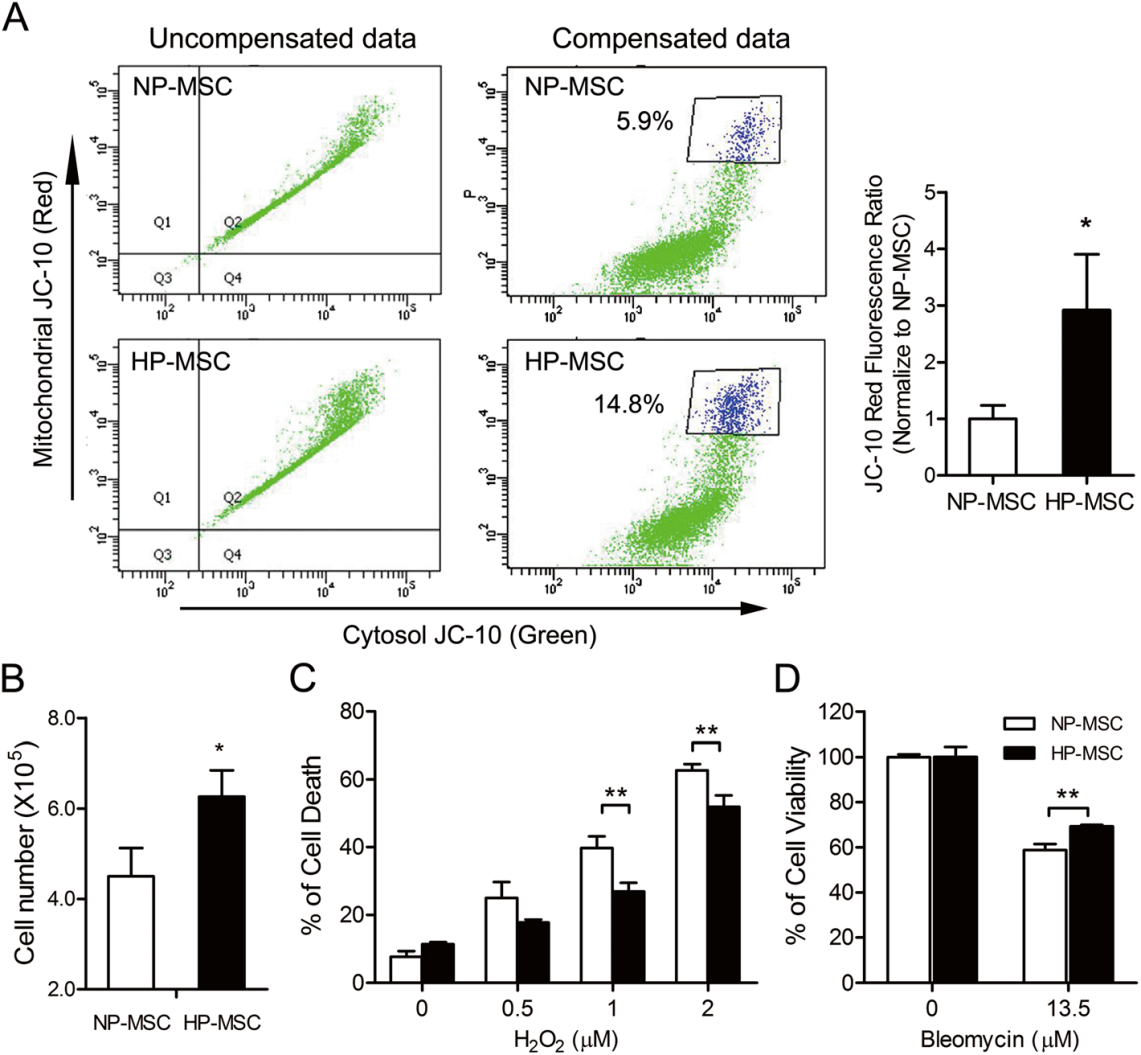
Hypoxic preconditioning promotes cell proliferation and decreases hydrogen peroxide- or belomycin-induced cell death in mesenchymal stem cells. **(A)** Mesenchymal stem cells (MSCs) were stained with 1 μM JC-10 for 30 minutes, and were then analyzed by flow cytometry. Owing to the dual wavelength emission of JC-10, most stained cells distributed at the double-positive region (top right quadrant). Electronic compensation was made to correct the bleed of the green (monomer) and red (aggregate) fluorescence signals into the FL2 and FL1 channels. Right column shows compensated data. The gated region represented the higher mitochondrial membrane potential cell population. The histogram on the right shows the quantification of three independent normoxia-preconditioned MSCs (NP-MSCs) and hypoxia-preconditioned MSCs (HP-MSCs) samples of JC-10 staining. **(B)** Cell number of NP-MSCs and HP-MSCs was determined by an automated cell counter. **(C)** Cell viability of NP-MSCs and HP-MSCs treated with the indicated H_2_O_2_ concentration for 1 hour as assessed by flow cytometric analysis with Annexin V/propidium iodide (PI) staining. Percentages of Annexin V/PI-double positive cells (dead cells population) are shown. **(D)** MTT assays of cell viability of bleomycin-treated mouse lung epithelial cells (MLE-12) in the presence of NP-MSC- or HP-MSC-conditioned medium for 48 hours. *P < 0.05, **P < 0.01, differences between HP-MSCs and the NP-MSC controls.

Mitochondria are critical oxygen sensors linked to various protective effects, such as enhancement of antioxidant defense, cell survival and anti-apoptosis [39,40]. To determine the mitochondrial functions in HP-MSCs in relation to NP-MSCs, the mitochondrial membrane potential in MSCs was assessed using membrane-permeant dual-emission potential-sensitive JC-10 dye and flow cytometry analysis. Dot plot revealed that all JC-10-stained cells distributed into the double-positive region (Figure 2A; left panel). Due to the dual wavelength emission of JC-10, electronic compensation was used to correct for spillage of the green (monomer) and red (aggregate) fluorescence signals into the FL2 and FL1 channels, respectively [41]. The compensated results showed a 14.8% higher red fluorescence signal in HP-MSCs when compared with that in NP-MSCs (approximately 5.9% higher red fluorescence signal), representing a 2.5-fold increment (Figure 2A; right panel).

### Hypoxic preconditioning promotes cell proliferation, reduces hydrogen peroxide-induced cytotoxicity in mesenchymal stem cells and attenuates bleomycin-induced alveolar epithelial cell death

The effects of hypoxic culture on MSC proliferation and expansion efficiency are dependent on the sources of MSCs, oxygen concentration, seeding density and the culturing duration of the MSCs [20]. MSCs cultured in hypoxic conditions increased by about 40% in cell number when compared to normoxic cells (cell number (×10^5^) of NP-MSCs = 4.50 ± 0.62, versus HP-MSCs = 6.27 ± 0.58; *P* < 0.01; Figure 2B). The hypoxic conditions used showed no adverse effects on cell viability (data not shown). To study the effects of hypoxic preconditioning on H_2_O_2_-induced cytotoxicity, HP-MSCs were cultured for 24 hours, followed by exposure to H_2_O_2_ for 1 hour. Results showed that H_2_O_2_ induced cell death in a dose-dependent manner, as measured by annexin V/PI staining (1 μM H_2_O_2_ treatment with NP-MSCs (39.67 ± 3.49) versus HP-MSCs (26.83 ± 2.62); 2 μM H_2_O_2_ treatment with NP-MSCs (62.70 ± 1.82) versus HP-MSCs (51.95 ± 3.29); Figure 2C). As anticipated, there were significant degrees of protection against H_2_O_2_-induced cell death in HP-MSCs compared with NP-MSCs (Figure 2C). Mouse alveolar epithelial cells (MLE-12) were further used to examine the cytoprotective effects of paracrine factors secreted by MSCs in attenuating BLM-induced cell death. MLE-12 cells were cultured in the presence of conditioned medium (CM) from NP-MSCs or HP-MSCs while under BLM treatment, followed by MTT assays. CM from HP-MSC cultures was able to improve lung epithelial cell survival compared to the control and NP-MSC CM during BLM treatment (58.70 ± 2.73 versus 69.27 ± 0.63, *P* < 0.01; Figure 2D).

### Hypoxia-preconditioned mesenchymal stem cell-conditioned medium attenuates extracellular matrix production through transforming growth factor-β1-mediated Akt signaling

**Figure 3:**
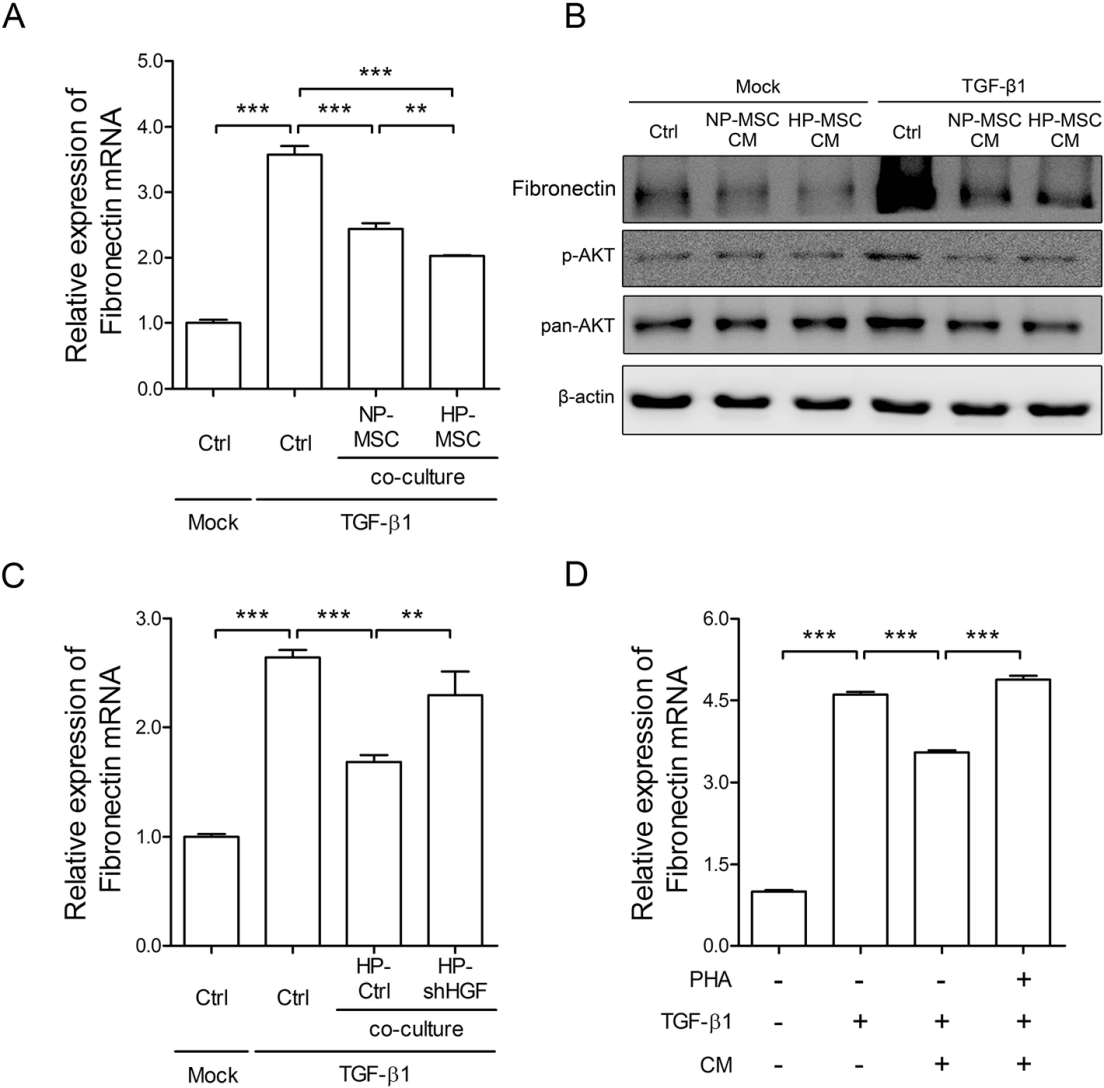
Hypoxia-preconditioned mesenchymal stem cells attenuate extracellular matrix production through transforming growth factor-β1-mediated Akt signaling. In the assessment of the anti-fibrotic effects of mesenchymal stem cells (MSCs), transforming growth factor (TGF)-β1-stimulated MRC-5 fibroblasts were used. **(A)** Co-culture experiments were performed in which mock control (Ctrl, culture medium only), TGF-β1 control, normoxia-preconditioned MSCs (NP-MSCs) or hypoxia-preconditioned MSCs (HP-MSCs) were seeded in the upper chamber, and TGF-β1 (1 ng/ml)-treated MRC-5 fibroblast cells were seeded in the lower chamber. After 24 hours incubation, quantitative real-time RT-PCR was performed to examine the fibronectin mRNA levels in MRC-5 cells. **(B)** Western blot analysis of fibronectin and phosphorylated Akt in TGF-β1-treated MRC-5 cells after incubation with MSC-conditioned media (CM) for 24 hours. **(C)** Co-culture experiments were performed in which mock Ctrl, TGF-β1 Ctrl, HP-Ctrl (hypoxia-pretreated MSCs transfected with control scramble short hairpin RNA (shRNA) plasmid) or HP-shHGF were seeded in the upper chamber, and TGF-β1 (1 ng/ml)-treated MRC-5 fibroblast cells were seeded in the lower chamber. After 24 hours incubation, quantitative real-time RT-PCR was performed to examine the fibronectin mRNA levels in MRC-5 cells. **(D)** MRC-5 cells were preincubated with or without PHA-665752 (1 μM) for 24 hours, and then cells were treated with or without HP-MSC CM for 24 hours. Quantitative real-time RT-PCR was performed to examine changes in the fibronectin mRNA levels in the MRC-5 cells. Values were normalized to the β-actin gene and are expressed relative to the control (Ctrl) group. **P < 0.01, ***P < 0.001. HGF, hepatocyte growth factor.

Previous studies have shown that TGF-β1 induces synthesis of ECM components, resulting in increased phosphorylation of Akt and its downstream signaling in fibroblast cells [42,43]. To assess the anti-fibrotic effects of MSCs, the production of the ECM protein fibronectin and downstream signaling in the TGF-β1-stimulated MRC-5 fibroblasts were examined using an *in vitro* co-culture system and treatment with CM. TGF-β1 treatment increased both the fibronectin mRNA (3.57 ± 013, expressed as relative gene expression changes) and protein expression levels in MRC-5 cells and also activated Akt signaling (Figure 3). As anticipated, ECM synthesis was significantly attenuated in both normoxic- and hypoxic-MSCs groups, while HP-MSCs showed higher inhibitory effects for ECM production than NP-MSCs (2.03 ± 0.01 after co-culture with HP-MSCs versus 3.57 ± 0.13 (media control) and 2.44 ± 0.08 (NP-MSCs), expressed as relative gene expression changes; Figure 3A). Western blot analysis also showed that CM collected from both normoxic- and hypoxic-MSCs downregulated the expression of phosphorylated AKT and fibronectin (Figure 3B).

Hence, we used two approaches to examine whether HP-MSCs attenuated ECM production through HGF. Firstly, genetic knock-down of HGF with short hairpins (shRNAs) in MSCs was used, followed by the use of an *in vitro* co-culture system to assess the anti-fibrotic effects. After 24 hours of co-culture, MSCs transfected with shRNA showed about a 50% decrease in the HGF mRNA level compared to cells transfected with the control scramble shRNA plasmid. Under hypoxic conditions, expression levels of HGF were significantly decreased in MSCs transfected with shRNA compared to the control group (data not shown). On the other hand, knockdown of HGF also abrogated the suppressive effects of HP-MSCs on TGF-β1-induced overexpression of fibronectin (2.64 ± 0.07 after co-culture with control media versus 1.68 ± 0.06 (HP-MSCs) and 2.29 ± 0.22 (HP-shHGF), expressed as relative gene expression changes; Figure 3C). Secondly, pharmacological inhibition of c-Met with its specific inhibitor, PHA-665752, to suppress HGF signaling in MRC-5 cells was also tested. Results showed that ECM production significantly increased in the MRC-5 cells treated with TGF-β1 plus PHA-665752. Although treatment with HP-MSC CM obviously suppressed expression of fibronectin, co-treatment with HP-MSC CM and PHA-665752 significantly inhibited the suppressive effects (3.55 ± 0.04 after treatment with HP-MSC CM versus 4.61 ± 0.05 (control media) and 4.88 ± 0.07 (HP-MSC CM plus PHA-665752), expressed as relative gene expression changes; Figure 3D). The results indicated that paracrine factors, such as HGF, derived from CM of HP-MSCs play a crucial role for attenuation of ECM production.

### Improvement of pulmonary respiratory functions of hypoxia-preconditioned mesenchymal stem cell treatment in the bleomycin-induced pulmonary fibrosis mouse model

**Figure 4:**
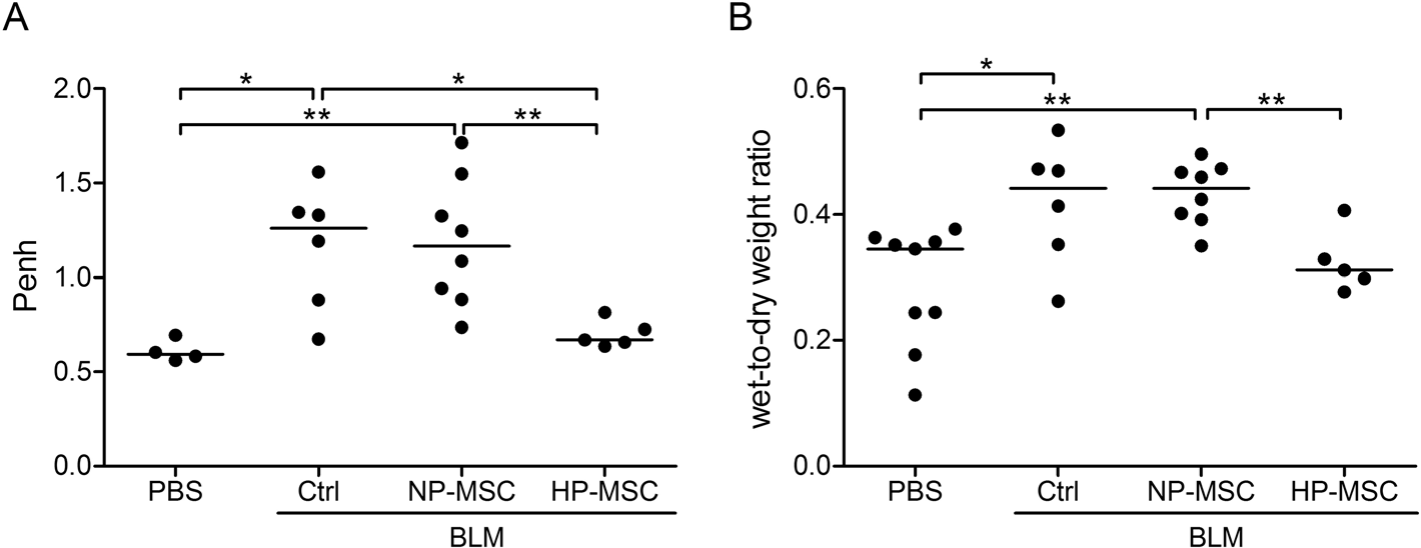
Hypoxia-preconditioned mesenchymal stem cell transplantation improves long-term attenuation of bleomycin-induced airway constriction and lung edema. **(A)** Pulmonary functions were evaluated as enhanced respiratory pause (Penh) values in animals that received normoxia-preconditioned mesenchymal stem cells (NP-MSCs), hypoxia-preconditioned mesenchymal stem cells (HP-MSCs) or phosphate-buffered saline (PBS) for 18 days following bleomycin (BLM) administration on day 3. Whole-body plethysmography was employed and Penh was used as a noninvasive index of airway dysfunction. **(B)** After sacrificing the mice on day 21, the lung wet-to-dry weight ratio was determined to assess pulmonary edema. Each dot represents an individual mouse with the mean shown for n ≥ 5 per group. *P < 0.05, **P < 0.01. Ctrl, control.

To evaluate the therapeutic efficacy of HP-MSCs in the BLM-induced pulmonary fibrosis mouse model, WBP was used to monitor lung functions of the treated mice. The respiratory parameter, in particular Penh, was measured as a noninvasive index of BLM-induced airway dysfunction [44]. In the experiments, the Penh values showed a two-fold increment at day 21 after BLM treatment compared with the PBS control group (0.61 ± 0.06 versus 1.16 ± 0.33, *P* < 0.05; Figure 4A). In addition, the BLM HP-MSC group had the lowest Penh value which was approximately the same as the PBS control; however, the BLM NP-MSC group showed no significant improvements in lung function in the fibrosis animal model (0.70 ± 0.07 in BLM HP-MSCs versus 1.19 ± 0.34 (BLM NP-MSCs) and 1.16 ± 0.33 (BLM control); Figure 4A). In addition, compared with the PBS control, the mice treated with BLM for 21 days showed a significant increment in the lung wet-to-dry ratio, indicating the degree of pulmonary edema caused by an inflammatory response in the lung. Likewise, the BLM HP-MSC group showed a lower wet-to-dry ratio than the BLM NP-MSC and BLM control groups at day 18 after stem cells transplantation (0.32 ± 0.05 in BLM HP-MSC group versus 0.43 ± 0.05 (BLM NP-MSC) and 0.42 ± 0.10 (BLM control); Figure 4B) also indicating that transplantation of HP-MSCs significantly reduced lung fluid content caused by BLM treatment. Taken together, our results indicated that transplanted HP-MSCs showed better therapeutic effects and improved lung functions in mice with BLM-induced pulmonary fibrosis.

### Downregulated expression of inflammatory and fibrotic factors in hypoxia-preconditioned mesenchymal stem cell-transplanted bleomycin-induced pulmonary fibrosis mouse model

**Figure 5:**
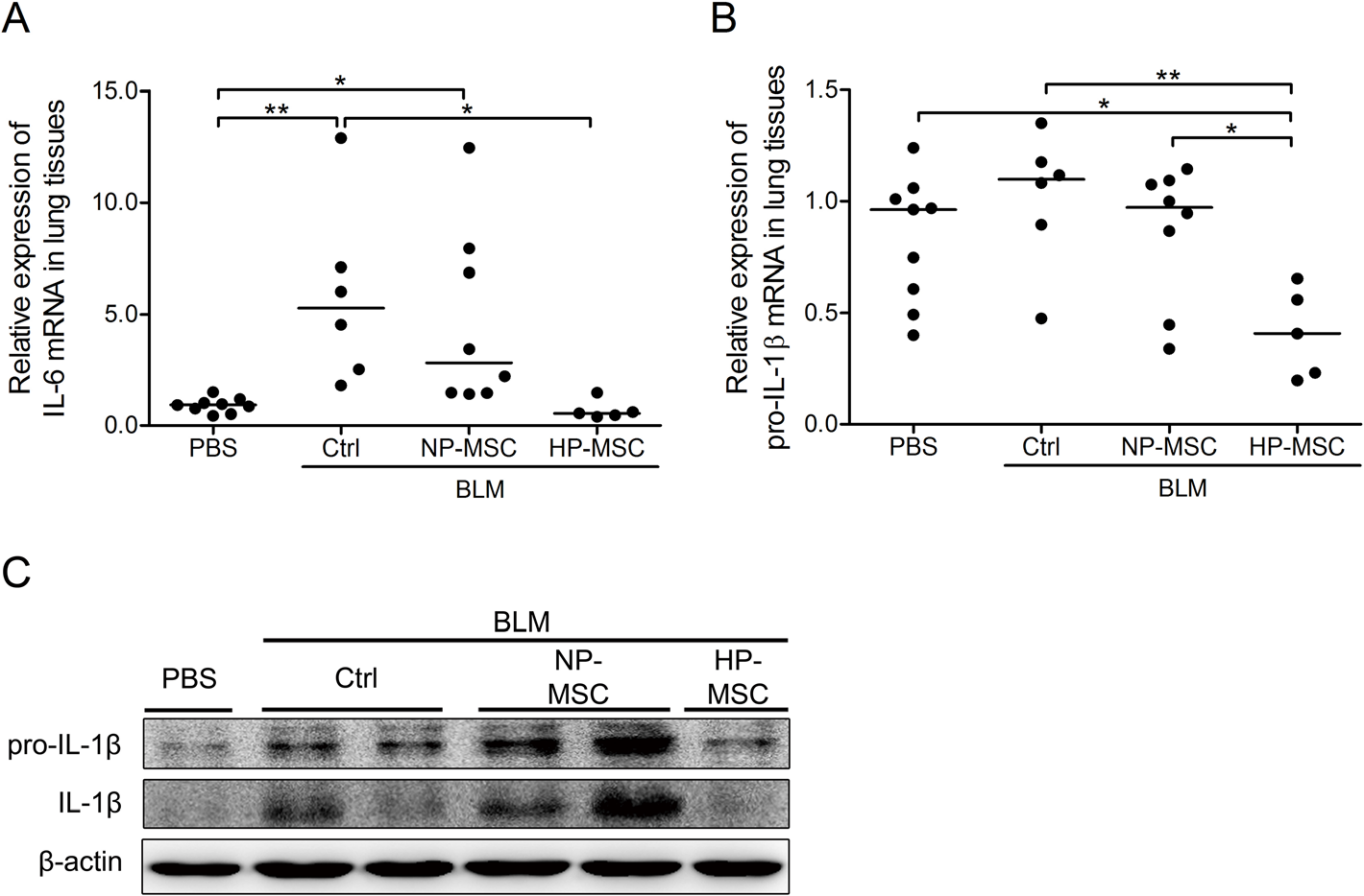
Hypoxia-preconditioned mesenchymal stem cell transplantation downregulates expression of inflammatory factors in the bleomycin-induced pulmonary fibrosis mouse model. Quantitative real-time RT-PCR was performed to analyze the expression of inflammatory factors **(A)** interleukin (IL)-6 and **(B)** pro-IL-1β in the lung tissues of animals that received normoxia-preconditioned mesenchymal stem cells (NP-MSCs), hypoxia-preconditioned mesenchymal stem cells (HP-MSCs) or phosphate-buffered saline (PBS) for 18 days following bleomycin (BLM) administration on day 3. Values are normalized to the GAPDH values and expressed relative to the PBS group. *P < 0.05, **P < 0.01. Each dot represents an individual mouse with the mean shown for n > 5 per group. **(C)** Western blot analysis of pro-IL-1β and IL-1β in whole lung tissues from each group. Ctrl, control.

**Figure 6:**
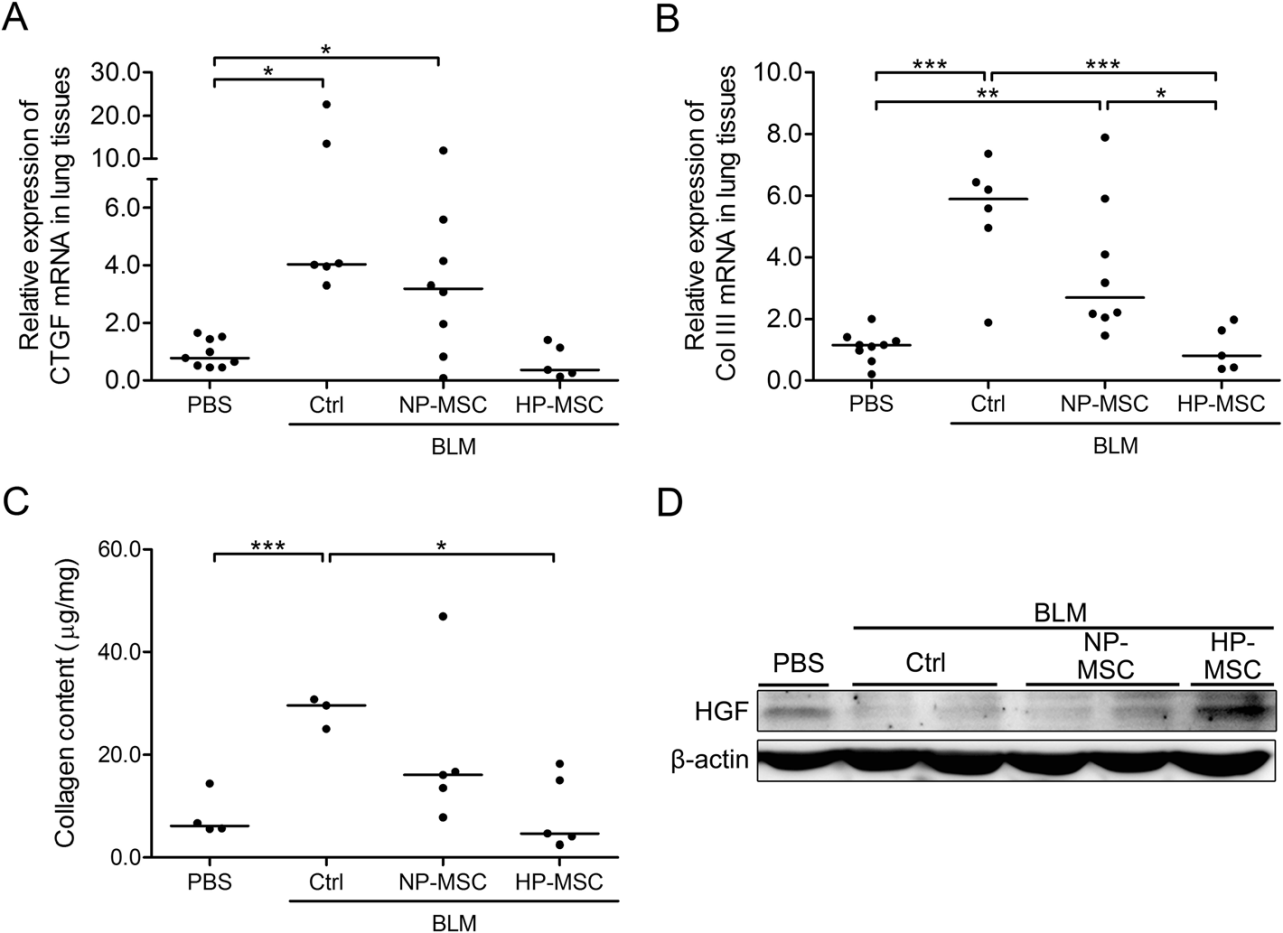
Hypoxia-preconditioned mesenchymal stem cell transplantation reduces pulmonary collagen deposition and fibrosis-related genes expression in the bleomycin-induced pulmonary fibrosis mouse model. **(A,B)** Quantitative real-time RT-PCR was performed to analyze the expression of fibrotic indicators **(A)** connective tissue growth factor (CTGF) and **(B)** collagen type III (Col III) in the lung tissues of mice that received normoxia-preconditioned mesenchymal stem cells (NP-MSCs), hypoxia-preconditioned mesenchymal stem cells (HP-MSCs) or phosphate-buffered saline (PBS) for 18 days following bleomycin (BLM) administration on day 3. Values are normalized to the GAPDH gene and are expressed relative to the PBS group. **(C)** Total collagen content of whole lung tissues from each group was determined by Sircol Collagen Assay. *P < 0.05, ***P < 0.001; n ≥ 5. **(D)** Western blot analysis of hepatocyte growth factor (HGF) in whole lung tissues from each group.

To determine the effects of HP-MSCs on inflammation and fibrosis in the BLM-induced pulmonary fibrosis model, expression levels of possible mediators that may be involved, including pro-inflammatory cytokine and fibrotic factors, were determined. The mRNA level of the inflammation-mediating IL-6 was significantly upregulated 21 days after BLM treatment compared with the PBS control group (0.91 ± 0.32 versus 5.81 ± 4.01, *P* < 0.01; Figure 5A). On the other hand, pro-IL-1β showed no significant upregulation at the mRNA level, but the protein levels of the precursor and mature IL-1β were markedly overexpressed (Figure 5B,C). However, there was a significant reduction in the expression of these inflammatory mediators in the HP-MSC transplantation group (IL-6 mRNA level: 0.69 ± 0.44 in BLM HP-MSC group versus 4.65 ± 4.04 (BLM NP-MSC) and 5.81 ± 4.01 (BLM control); pro-IL-1β mRNA level: 0.41 ± 0.20 in BLM HP-MSC group versus 0.86 ± 0.30 (BLM NP-MSCs) and 1.02 ± 0.30 (BLM control); Figure 5A-C). Interestingly, there was no discernible difference in the levels of inflammation between the BLM NP-MSCs and BLM control groups (Figure 5A-C). Furthermore, expression of two factors mediating fibrosis, namely collagen type III and connective tissue growth factor (CTGF), was significantly upregulated at day 21 post-BLM treatment, but expression of collagen type III and CTGF was clearly reduced on HP-MSC transplantation (collagen type III mRNA level: 1.04 ± 0.72 in BLM HP-MSC group versus 3.62 ± 2.24 (BLM NP-MSC) and 5.40 ± 1.90 (BLM control); CTGF mRNA level: 0.66 ± 0.58 in BLM HP-MSC group versus 3.86 ± 3.70 (BLM NP-MSC) and 8.58 ± 7.91 (BLM control); Figure 6A,B). Moreover, there was clearly collagen deposition after BLM treatment, but only low collagen content could be detected in the HP-MSC transplanted mice which was comparable to the normal level (8.87 ± 7.20 μg/mg wet lung tissue in BLM HP-MSC group versus 20.17 ± 15.36 (BLM NP-MSCs) and 28.48 ± 3.06 (BLM control) and 8.02 ± 4.26 (PBS control); Figure 6C). Noting the downregulation of inflammatory and fibrotic mediators, other possible cytoprotective mediators were further investigated by examining the expression levels of HGF in whole-lung tissues by western blot analysis. HGF showed decreased levels in the BLM only and MSC-treated mice, but was overexpressed in the HP-MSC-transplanted mice (Figure 6D). Taken together, transplantation of HP-MSCs exerted better inhibitory effects on upregulating pro-inflammatory and fibrotic factors and on inducing the accumulation of collagen than in the BLM NP-MSC group (Figures 5 and 6).

### Hypoxia-preconditioned mesenchymal stem cell transplantation alleviates histological changes in the lungs of the bleomycin-induced pulmonary fibrosis mouse model

**Figure 7:**
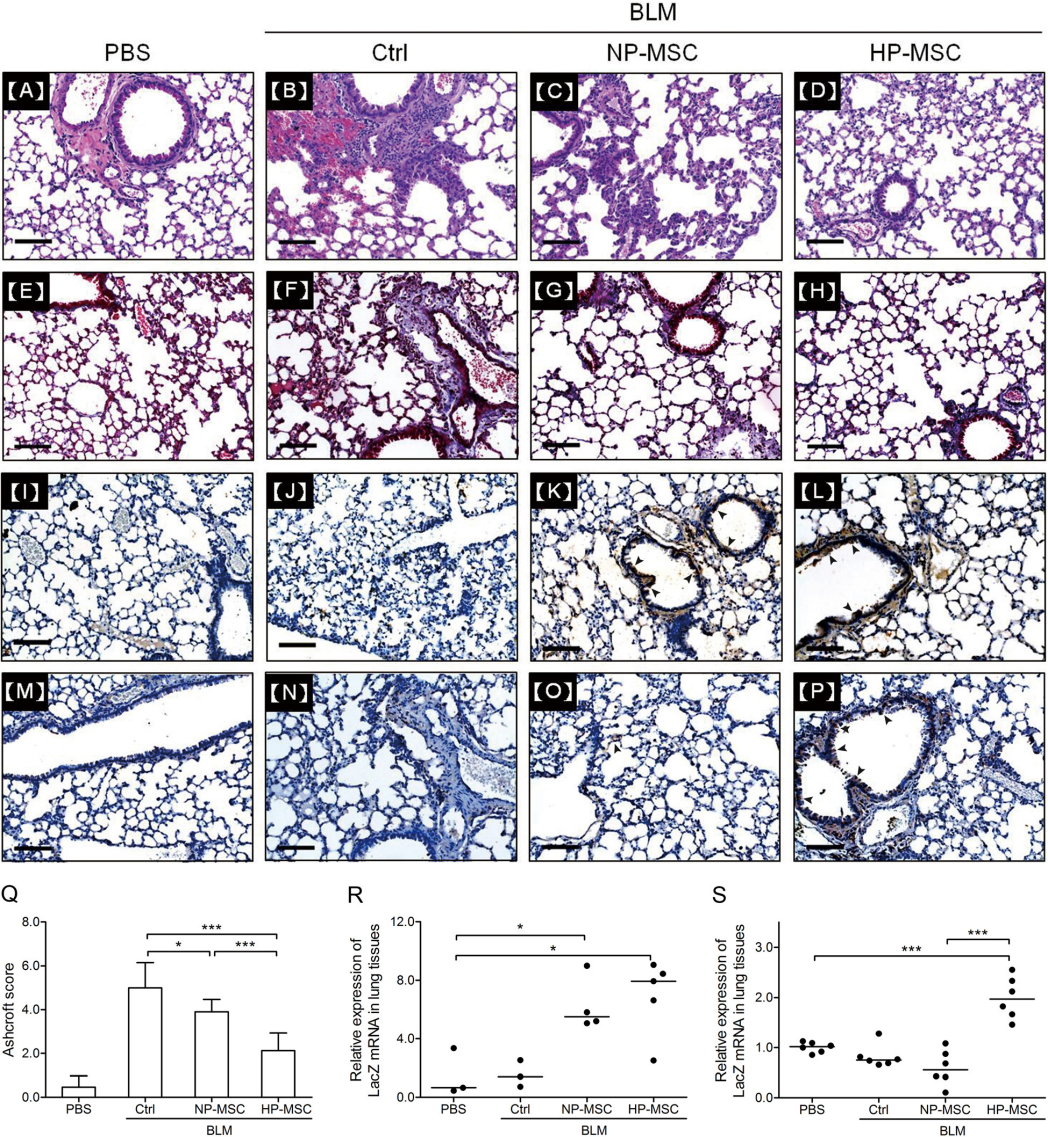
Hypoxia-preconditioned mesenchymal stem cell transplantation reduces fibrotic histopathologic changes in the bleomycin-induced pulmonary fibrosis mouse model. Representative images at day 21 of **(A-D)** hematoxylin and eosin and **(E-H)** Masson’s trichrome-stained histological sections from each group. Immunohistochemistry staining was performed to observe the distribution of LacZ in lung tissues at **(I-L)** day 7 and **(M-P)** day 21 of mesenchymal stem cell (MSC)-treated mice. (A,E,I,M) Phosphate-buffered saline (PBS) control, (B,F,J,N) bleomycin (BLM) + PBS, (C,G,K,O) BLM + normoxia-preconditioned MSCs (NP-MSCs), (D,H,L,P) BLM + hypoxia-preconditioned MSCs (HP-MSCs). Scale bar = 100 μm. Solid arrows around the bronchi indicate LacZ-positive cells (brown spots). **(Q)** The fibrotic changes in the lung were quantified by using Ashcroft scores on day 21 after administration, ranging from 0 (normal lung) to 8 (complete fibrosis). Quantitative real-time RT-PCR was performed to detect the expression of LacZ in the lung tissues of mice that received NP-MSCs, HP-MSCs or PBS on **(R)** day 4 and **(S)** day 18 following BLM administration on day 3. Values are normalized to the GAPDH values and expressed relative to the PBS group. *P < 0.05, ***P < 0.001. Each dot represents an individual mouse with the mean shown for n ≥ 3 per group. Ctrl, control.

The lung histopathologic sections from each experimental group at day 18 after stem cell transplantation are shown in Figure 7. There was no obvious lesion or inflammatory infiltration in the lung of the PBS control group (Figure 7A). In the BLM-only group, hematoxylin and eosin and Masson’s trichrome staining showed that the pulmonary alveolus cavities were obviously decreased in size, the alveolar wall was thickened, and there was accumulation of inflammatory cells (Figure 7B,F). Lungs from MSC-engrafted mice showed thinner alveolar wall, but with accumulation of inflammatory cells (Figure 7C,G). On the other hand, lung tissues of HP-MSC-transplanted mice showed a significant reduction of the alveolar wall thickness and accumulation of collagen in the lung interstitium (Figure 7D,H). Quantitative analysis was performed using the Ashcroft score (2.13 ± 0.81 (BLM NP-MSC) versus 3.90 ± 0.57 (BLM NP-MSC) and 5.0 ± 1.15 (BLM control), *P* < 0.001; Figure 7Q). To determine whether engrafted HP-MSCs survived and adopted in the damaged lungs at day 7 and 21 after BLM treatment, we intratracheally administered LacZ reporter-transduced MSCs (β-Gal-MSCs) into the BLM-induced pulmonary fibrosis mouse model. LacZ expression was determined by immunohistochemical staining and quantitative real-time RT-PCR. At day 4 after stem cell engraftment (Figure 7I-L,R), there were no LacZ-labeled cells or LacZ mRNA observed in lung tissue sections of either the PBS or BLM control group. However, an approximately six- to eight-fold increment of LacZ mRNA was detected and numerous LacZ-labeled cells were observed to be distributed around the bronchi in both the NP-MSC and HP-MSC treatment groups (Figure 7K,L,R). As anticipated, at day 18 after stem cell transplantation (Figure 7M-P,S), there were no LacZ-labeled cells or LacZ mRNA observed in lung tissue sections of either the normal, injured or NP-MSC group. However, an approximately three-fold increment of LacZ mRNA was detected and numerous LacZ-labeled cells were observed and distributed around the bronchi in the HP-MSC treatment group (Figure 7P,S).

Our data indicated that hypoxic preconditioning enhanced the survival rate of engrafted MSCs, exerted better therapeutic effects and improved lung functions in BLM-induced pulmonary fibrotic mice.

## Discussion

Accumulating evidence suggests that stem cell-based therapies may be used for lung regeneration and modulation of inflammatory and fibrotic processes [7]. Ortiz and colleagues [45] first reported that systemically engrafted MSCs possessed the ability to ameliorate fibrotic effects in mice challenged with BLM. Other reports have shown that multiple mechanisms are probably involved in the engrafted MSCs leading to improved outcome in animal models with pulmonary fibrosis [7,46]. In this work, we are the first to demonstrate that engraftment of HP-MSCs rendered better therapeutic effects than untreated MSCs in a BLM-induced pulmonary fibrosis animal model. Hypoxic preconditioning upregulated cytoprotective and regenerative genes, stabilized mitochondrial membrane potentials, promoted cell proliferation and acted against H_2_O_2_-induced cell death in the treated MSCs. Moreover, HP-MSCs attenuated BLM-induced cell apoptosis and ECM production through TGF-β1-mediated Akt signaling via paracrine effects. HP-MSC engraftment improved lung functions, reduced lung edema and the levels of pro-inflammatory and fibrotic factors.

Although recent studies indicated that both local (intratracheal) and systemic (intravenous or intraperitoneal) infusion may be effective in attenuating lung injury, the optimal route of delivery remains unclear [47]. On the other hand, systemic administration of MSCs showed some difficult conditions for application to the clinical setting, such as the recipient requiring total body irradiation to promote stem cell engraftment [10]. Alternatively, considering the advantages of local administration to target the lung tissue, several recent studies reported that a lower dosage of stem cells are needed [48,49] through the intratracheal route and showed more effective airway regeneration [50,51] compared with the intravenous route.

Although MSC-based cell therapy has shown great potential in cell repair, the therapeutic effects of MSC-based approaches have met a bottleneck following transplantation. Leblond and colleagues [10] reported that a large number of the engrafted cells were trapped in the damaged lungs during short-term treatment. Less than 5% of implanted cells remained detectable in the injured lungs 7 days after transplantation [52]. Our results also showed that the number of engrafted MSCs dramatically decreased with time after transplantation (data not shown). To resolve poor therapeutic efficacy of long-term MSC transplantation, recent studies have proposed various strategies aiming to precondition implanted MSCs by sublethal physical and chemical stresses or genetic and pharmacological stimuli to increase tolerance of the transplanted cells to withstand the severe microenvironment of the transplanted sites [11-13,53].

Murry and colleagues [54] first described the phenomenon termed “ischemic preconditioning” which attenuated myocardium damage induced by ischemia. Our data suggest that hypoxic preconditioning may be a better solution for the transplanted cells to adapt to low oxygen tension microenvironment, or in organs with lesions, through systematically activating and altering a series of cellular responses [55-57]. Many of these alterations, including improved growth kinetics, homing ability, survival, regenerative, and genetic stability of the implanted cells are characterized by upregulation of cytoprotective genes HIF-1α, erythropoietin receptor, B-cell lymphoma 2, HGF and VEGF [21,40,58-60]. In addition, these cytoprotective factors exert both autocrine and paracrine effects [61]. In agreement with previous studies, our *in vitro* studies showed that HP-MSCs upregulated expression of cytoprotective genes (Figure 1). The reason why 1.5% O_2_ concentration was chosen in this study was that 1.5% mimics the low oxygen concentration (1 to 2%) naturally encountered by MSCs in their bone marrow niche [62-64]. It has been reported that short-term (24 hour) exposure of MSCs to hypoxia at 1.5% O_2_ stabilizes the HIF-1 protein, and clearly promotes proliferation, cell cycle progression and migration [65]. The viability of MSCs was not adversely affected by 24 hours of hypoxia at 1.5% O_2_ (data not shown). The mRNA expression level of HIF-1α reached the peak level at 12 hours of hypoxic exposure (Figure 1A), whereas the HIF-1α protein level peaked after 24 hours of exposure (Figure 1E). On the other hand, western blot analyses showed that hypoxic conditions caused a time-dependent increase in the levels of the cytoprotective proteins (Figure 1), reaching maximum at 24 hours after hypoxic preconditioning.

The strategies of HP-MSC transplantation have shown potential benefits in several experimental disease models in acute kidney injury [30] and ischemic diseases [25,27,60,66]. Studies showed that therapeutic effects were improved on hypoxic treatment due to activation of HIF-1α followed by upregulation of growth factors and downregulation of pro-inflammatory cytokines and chemokines in hypoxic MSCs [59]. Such events positively support HP-MSCs to overcome limitations and circumvent hindrances in cell-based therapy [55]. Here, we found that intratracheal instillation of HP-MSCs provided better therapeutic effects than untreated MSCs after BLM exposure, and protected lung tissues from injuries by improving the pulmonary functions (Figure 4) and reducing the extent of inflammation (Figure 5) and fibrosis (Figure 6).

Mounting evidence has shown that BLM as a chemotherapeutic agent has undesirable side-effects in inducing lung injuries and triggering apoptosis of alveolar epithelial cells [67]. In addition, either BLM or downstream TGF-β1 may induce ECM synthesis, and ECM deposition is mediated through Akt signaling in fibroblasts [43]. Hence, dysregulation of intercellular cross-talk between alveolar epithelia and fibroblasts is a critical event in the fibrosis process. The net balance is disturbed when alveolar epithelial cells die and then factors such as TGF-β1 shift the balance toward enhancement of fibroblast proliferation and ECM production [68]. Our study showed that HP-MSCs possessed better cytoprotective abilities to resist stimuli-induced alveolar epithelial cell death (Figure 2B,C) and to attenuate Akt-mediated ECM production in fibroblast cells through paracrine effects (Figure 3). Consistent with other studies, transplantated MSCs site-specifically migrate into damaged alveolar epithelial cells, and reduced alveolar epithelial cell apoptosis [45,69], resulting in the differentiation of MSCs into alveolar epithelial cells [70] through paracrine mechanisms [71]. Hence, transplantation of HP-MSCs inhibited production of pro-inflammatory mediators (Figure 5), attenuated ECM deposition (Figure 6C) and reduced alveolar wall thickness (Figure 7D) in the fibrosis lung model.

Many reports have shown some benefits from using MSCs to attenuate inflammatory response and fibrosis. However, the therapeutic efficacy may be affected by the transplanted cell number, cell ages, timing of transplantation, and treatment period [72]. Most studies assessed the therapeutic effects at day 7 or day 14 after BLM treatment, but only a few studies showed the longer period of treatment effects. Aguilar and colleagues noted that delivery of MSCs at the time of BLM injury and 3 days later, the end point of their experiments found that MSCs showed no effect in attenuating the ECM deposition and histological damage [73]. Moodley and colleagues noted that there was no change in aberrant lung collagen deposition in the MSC treatment group at day 21 after BLM injury [74]. Moreover, Cargnoni and colleagues noted that transplantation of MSCs slightly modulated lung inflammation during long-term treatment [75]. Similar results were shown in our study; NP-MSC treatment showed slight suppression of ECM deposition and no effect on inflammatory response at day 21 after BLM treatment. Therefore, HP-MSCs were more effective at reducing inflammatory and ECM production (Figures 4, 5 and 6).

Recent studies have demonstrated that HGF plays a key role in preventing fibrosis or scar formation after injuries [76,77]. Moreover, HGF exerts multiple protective effects on injured tissues via mitogenic, motogenic, anti-apoptosis, and anti-inflammatory and anti-fibrogenic signals [78-82]. Reports also showed that TGF-β1-induced fibrosis could be prevented by treatment with HGF through inhibiting myofibroblast differentiation [83,84] and ECM production [85-87]. In agreement with this reported evidence, the regenerative factor HGF was significantly upregulated in the HP-MSC-treated fibrotic lungs in the present study (Figure 6D).

## Conclusions

In summary, we report here that transplantation of HP-MSCs improves pulmonary functions and reduces inflammatory and fibrotic mediators in a BLM-induced pulmonary fibrosis model. The upregulation of HGF might play a key role in mediating the therapeutic effects of transplanted HP-MSCs.

## Abbreviations

BLM: bleomycin
CM: conditioned medium
CTGF: connective tissue growth factor
ECM: extracellular matrix
HGF: hepatocyte growth factor
HIF: hypoxia-inducible factor
HO-1: heme oxygenase 1
HP-MSC: hypoxia-preconditioned mesenchymal stem cell
IL: interleukin
IPF: idiopathic pulmonary fibrosis
MSC: mesenchymal stem cell
NP-MSC: normoxia-preconditioned mesenchymal stem cell
PBS: phosphate-buffered saline
Penh: enhanced respiratory pause
PI: propidium iodide
shRNA: short hairpin RNA
TGF: transforming growth factor
VEGF: vascular endothelial growth factor
WBP: whole-body plethysmography
